# Effect of a figure-of-eight cerclage wire with two Kirschner wires on fixation strength for transverse metacarpal shaft fractures: an in vitro study with artificial bone

**DOI:** 10.1186/s12891-021-04276-8

**Published:** 2021-05-10

**Authors:** Yung-Cheng Chiu, Cheng-En Hsu, Tsung-Yu Ho, Yen-Nien Ting, Ming-Tzu Tsai, Jui-Ting Hsu

**Affiliations:** 1 School of Medicine, China Medical University, Taichung, 404 Taiwan; 2 Department of Orthopedic Surgery, China Medical University Hospital, Taichung, 404 Taiwan; 3 Department of Orthopaedics, Taichung Veterans General Hospital, Taichung, 407 Taiwan; 4 Sports Recreation and Health Management Continuing Studies-Bachelor’s Degree Completion Program, Tunghai University, Taichung, 407 Taiwan; 5 3D Printing Medical Research Center, China Medical University Hospital, Taichung, 404 Taiwan; 6 Department of Biomedical Engineering, Hungkuang University, Taichung, 433 Taiwan, ROC; 7 School of Dentistry, College of Dentistry, China Medical University, Taichung, 404 Taiwan; 8 Department of Bioinformatics and Medical Engineering, Asia University, Taichung, 413 Taiwan

**Keywords:** Metacarpal shaft fracture, K-wire, Figure-of-eight cerclage wire

## Abstract

**Background:**

Metacarpal shaft fractures are a common type of hand fracture. Despite providing strong fixation strength, plate fixation has numerous shortcomings. Concerning internal fixation with Kirschner wires (K-wires), although this approach is frequently used to treat metacarpal shaft fractures, the lack of functional stability may result in fixation failure.

**Objective:**

To evaluate the effect of figure-of-eight cerclage wire on fixation for transverse metacarpal shaft fractures using two K-wires.

**Materials and methods:**

We used a saw blade to create transverse metacarpal shaft fractures in 14 fourth-generation artificial third metacarpal bones (Sawbones, Vashon, WA, USA), which were assigned to groups undergoing fixation with two K-wires (KP) or with two K-wires and figure-of-eight cerclage wire (KP&F8). All specimens were subjected to material testing, specifically cantilever bending tests. The maximum fracture force and stiffness of the two fixation types were determined on the basis of the force–displacement data. The Mann–Whitney *U* test was used to compare between-group differences in maximum fracture force and stiffness.

**Results:**

The maximum fracture force of the KP group (median ± interquartile range = 97.30 ± 29.70 N) was significantly lower than that of the KP&F8 group (153.2 ± 69.50 N, *p* < 0.05; Figure 5a), with the median of the KP&F8 group exceeding that of the KP group by 57.5%. Similarly, stiffness was significantly lower in the KP group (18.14 ± 9.84 N/mm) than in the KP&F8 group (38.25 ± 23.49 N/mm; *p* < 0.05; Figure 5b), with the median of the KP&F8 group exceeding that of the KP group by 110.9%.

**Conclusion:**

The incorporation of a figure-of-eight cerclage wire increased the maximum fracture force and stiffness by 57.5 and 110.9%, respectively, compared with those achieved in standard two K-wire fixation. Therefore, hand surgeons are advised to consider the proposed approach to increase fixation strength.

## Introduction

Metacarpal fractures account for 36–42% of hand fracture injuries [1]. Metacarpal neck fractures are the most common type of metacarpal fracture [2]. Although less common, metacarpal shaft fractures are more difficult to treat because the metacarpal shafts are primarily composed of cortical bones, whereas the metacarpal neck mostly consists of cancellous bone. Therefore, metacarpal shaft fractures require surgical implantation to provide strong fixation strength and to facilitate bone union [3]. Variations in injury mechanisms can lead to different types of metacarpal shaft fractures, such as transverse, oblique, spiral, or comminuted fractures. Conservative treatment with cast immobilization is only applicable to stable fractures in which the fractured bone is not displaced [4, 5]. Otherwise, surgical fixation is required. Transverse metacarpal shaft fractures are considered particularly unstable because of the small contact area of the fractured site and the exposure to traction force generated by the interosseous muscles [4]. Cast immobilization for transverse metacarpal shaft fractures can eventually result in fracture displacement and treatment failure because of insufficient force for maintaining fracture reduction [6, 7]. Accordingly, most relevant studies and hand surgeons’ recommendations are surgical reduction and metallic implant fixation for transverse metacarpal shaft fractures to optimize treatment results [4, 6].

In clinical settings, Kirschner wires (K-wires) or bone plates are typically used in the fixation of metacarpal shaft fractures [8, 9]. No consensus has been reached regarding which is more suitable for this purpose [7, 9, 10]. Compared with K-wires, bone plates have substantially higher biomechanical strength. However, the applicability of bone plates, including locking plates, for metacarpal shaft fracture fixation remains a topic of intense debate [3, 9, 11]. Moreover, numerous studies have identified the shortcomings of this approach for this procedure. For example, plate fixation requires surgical incisions, whereas K-wires can be positioned through minimally invasive surgery. Other drawbacks of plate fixation include postoperative metacarpophalangeal joint stiffness, extensor tendon adhesions, and iatrogenic injuries to the cutaneous nerves, all of which require secondary surgery for plate removal. This makes plate fixation much more expensive than K-wire fixation is [4, 12, 13]. Although using K-wires for the fixation of metacarpal shaft fractures is minimally invasive and relatively affordable, their fixation strength remains questionable [14]. From the biomechanical perspective, using only K-wires for fixation is associated with unsatisfactory bending and torque forces [11]. Nonetheless, numerous studies have reported lower incidences of extensor tendon adhesions and metacarpophalangeal joint stiffness with K-wire fixation than with plate fixation [15, 16]. In an effort to retain its strengths and compensate for its disadvantages, the present study developed a new fixation approach involving the placement of two K-wires and figure-of-eight cerclage wire.

Fixation involving the use of two K-wires and figure-of-eight cerclage wire was first implemented in the treatment of transverse patellar fractures [17]. Its satisfactory outcomes have led to its wide application in the treatment of various extremity fractures, including distal clavicle fractures [18], olecranon fractures [19], and medial malleolus fractures [20]. The advantages of this approach over K-wire fixation alone are as follows: (1) provision of greater antirotational force, (2) provision of greater anti-bending force, and most importantly, (3) the ability to convert tensile force into compressive force at the tension side cortex [21]. Specifically, this approach is effective in facilitating bone union when it is applied to the tension side of the fractured site.

Metacarpal shaft fractures are a common type of hand fracture. Although it provides strong fixation strength, plate fixation has numerous shortcomings. Internal fixation with K-wires is frequently used to treat metacarpal shaft fractures, but their lack of functional stability may result in fixation failure. In our previous study, [15] achieved satisfactory outcomes in the placement of two K-wires and figure-of-8 cerclage wire for the fixation of metacarpal neck fractures. The objective of the present study was to evaluate the effect of incorporating a figure-of-eight cerclage wire with two K-wires on fixation strength for transverse metacarpal shaft fractures.

## Materials and methods

### Specimen preparation

**Fig. 1:**
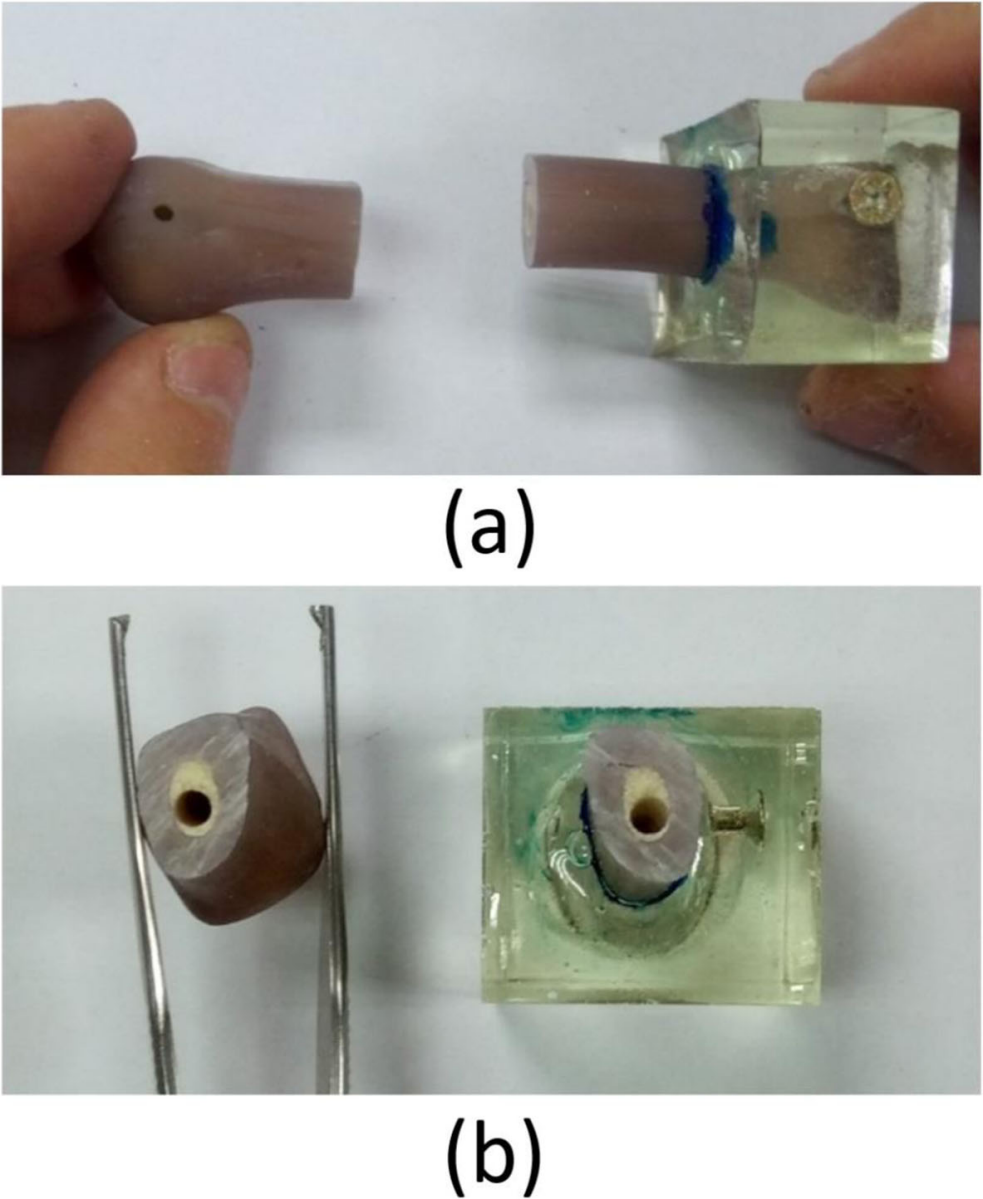
Artificial metacarpal bones with transverse shaft fractures. **a** Lateral view. **b** Cross-sectional view

Because of the challenge in obtaining a sufficient number of real metacarpal bones, the present study used 14 artificial fourth-generation third metacarpal bones (Sawbones, Vashon, WA, USA). The elastic modulus and density of the synthetic bone, which consisted of cortical and cancellous bone, were comparable to those of human bone (Fig. 1). The bone also included an artificial medullary canal. A metacarpal shaft fracture was made in each of the specimens using a miniature saw. The fracture was 30 mm from the distal articular surface. An industrial screw was inserted into the proximal side of the artificial bone and affixed with epoxy to enhance the strength. Moreover, the proximal end of each specimen was held in a custom fixture using epoxy clamps.

### Fixation approaches

**Fig. 2:**
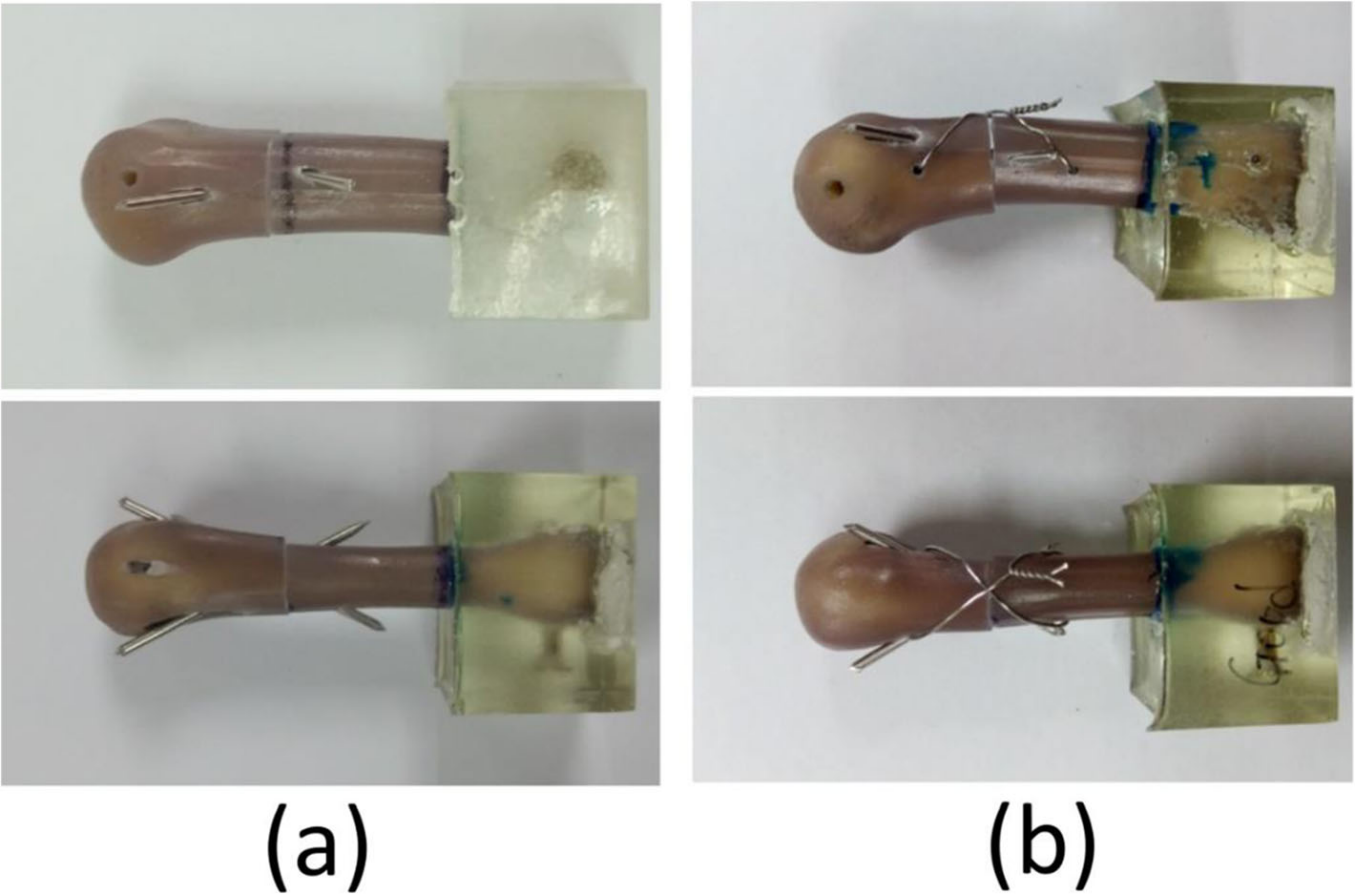
Artificial metacarpal bones with transverse shaft fracture subjected to fixation. **a** Two K-wire fixation. **b** Two K-wire fixation with a figure-of-eight cerclage wire. The top and bottom images present the lateral and dorsal views, respectively

**Fig. 3:**
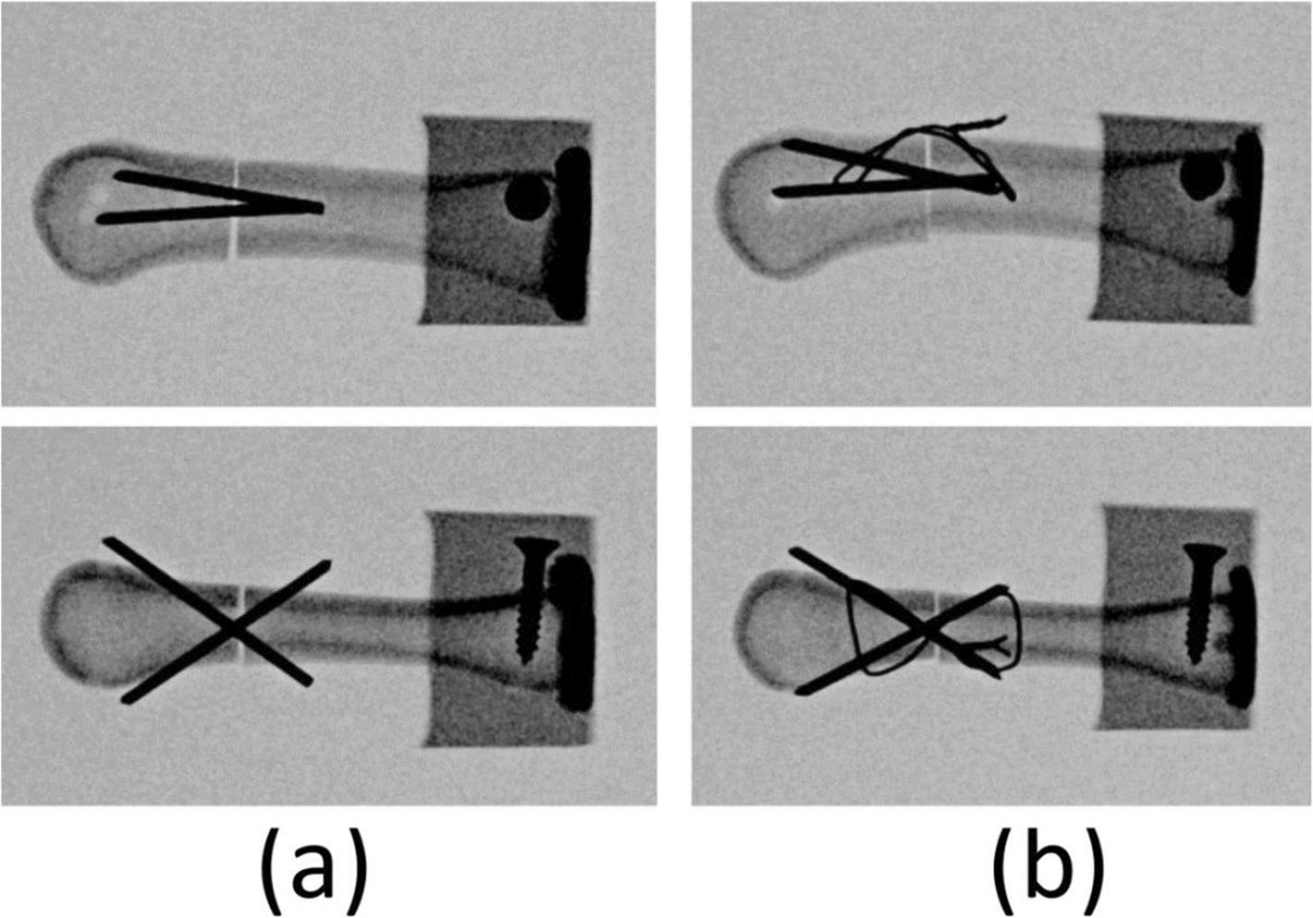
Radiographs of the specimens. **a** Two K-wire fixation. **b** Two K-wire fixation with a figure-of-eight cerclage wire. The top and bottom images present the lateral and dorsal views, respectively

The specimens were assigned to undergo fixation, which was performed by a single senior hand surgeon, Yung-Cheng Chiu, through one of two approaches, described as follows:

- The two K-wire (KP) group: The seven specimens were stabilized with two K-wires 1.5 mm in diameter, which were distally drilled from the dorsomedial and dorsolateral side of the metacarpal shaft and inserted through the fracture site; subsequently, they were proximally passed through the proximal volar cortex for cross-wire fixation. During surgery, fracture reduction was maintained using manual axial compression (Figs. 2a and 3a).
- The two K-wires with figure-of-eight cerclage wire (KP&F8) group: The seven specimens were stabilized with two K-wires 1.5 mm in diameter, which were distally drilled from the dorsomedial and dorsolateral side of the metacarpal shaft and inserted through the fracture site; subsequently, they were proximally passed through the proximal volar cortex for cross-wire fixation. During surgery, fracture reduction was maintained using manual axial compression. Next, two holes were transversely drilled approximately 1.5 cm from the fracture site in the proximal and distal bone fragment along the mid-lateral axis of the bone. A 25-gauge stainless steel wire was then passed through the bone tunnel and tightened into a figure-of-eight shape (Figs. 2b and 3b). When this procedure is performed in vivo, the wire should be placed under the extensor tendon, and the wire knot should be tied on the lateral side and moved to the lateral volar aspect of the metacarpal bone to prevent tendon injury. In essence, the extensor tendon must be treated gently during the procedure to prevent iatrogenic injury.

### Biomechanical test

**Fig. 4:**
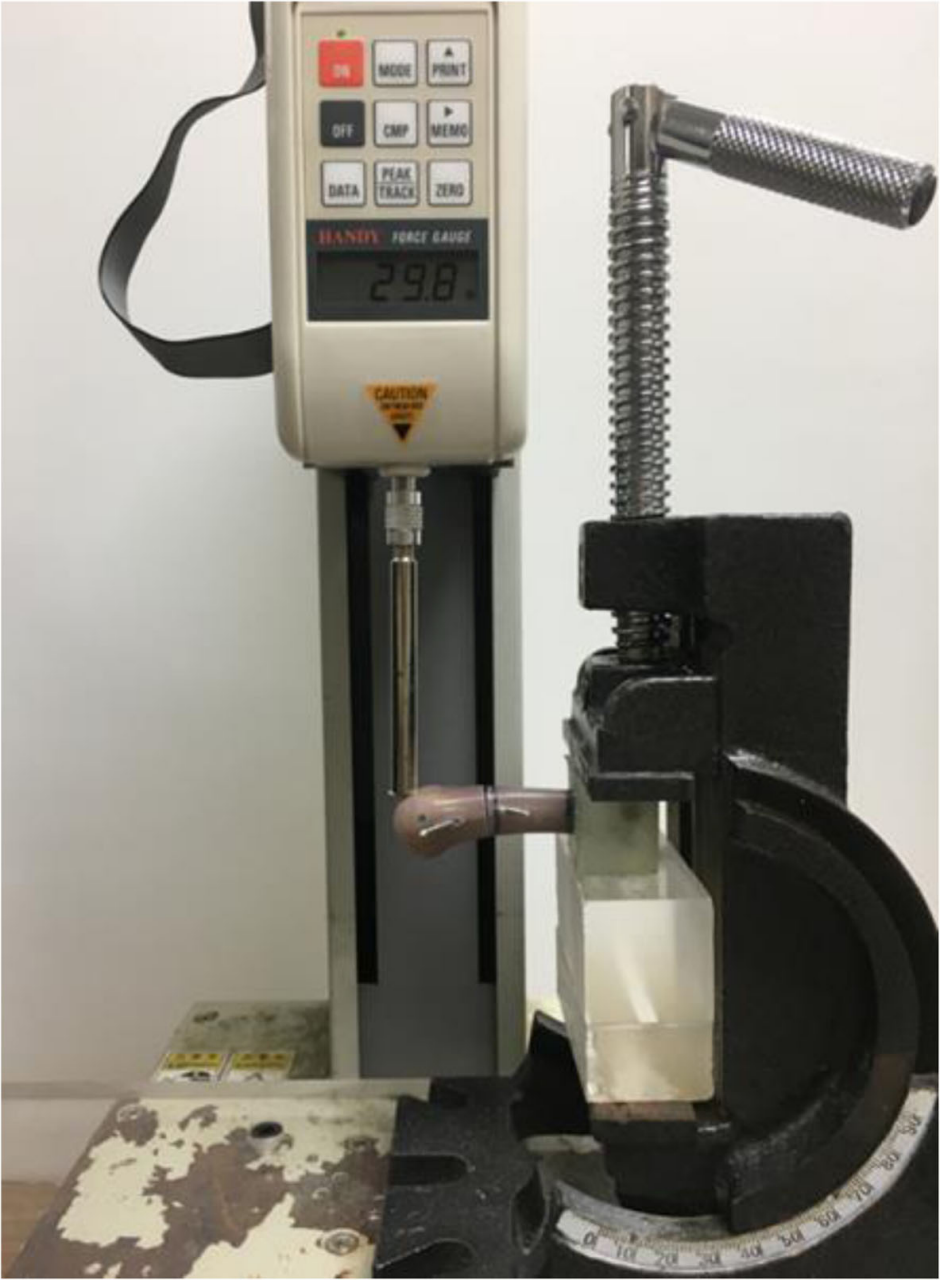
Experimental setup for the cantilever bending tests

With reference to previous studies [15, 22, 23], cantilever bending tests were performed to determine the fixation strength. The tests were conducted at a loading rate of 10 mm/min using a material testing system (JSV-H1000, Japan Instrumentation System, Nara, Japan; Fig. 4). A perpendicular load was applied to the dorsal side of each specimen 50 mm from the fixture until failure. The force–displacement data were recorded, and maximum fracture force and stiffness were determined for each specimen.

### Statistical analysis

The values of maximum fracture force and stiffness are presented as medians and interquartile ranges (IQRs). The Mann–Whitney *U* test was used to examine between-group differences in these two measures. All analyses were performed using IBM SPSS Statistics for Windows, version 19 (IBM Corp., Armonk, NY, USA). A *p* value < 0.05 was considered statistically significant.

## Results

**Fig. 5:**
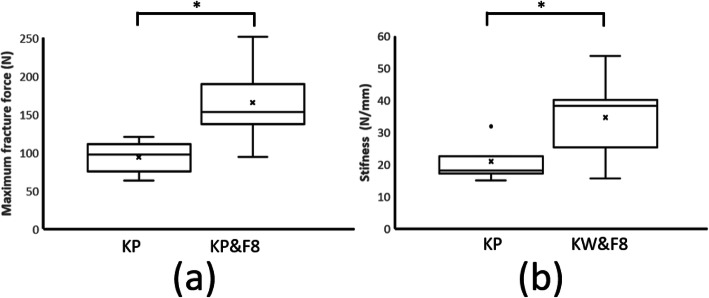
Box plot showing the **a** maximum fracture force and **b** stiffness of the specimens. *p < 0.05

Table 1 presents the results of the maximum fracture force and stiffness tests. The maximum fracture force of the KP group (97.30 ± 29.70 N) was significantly lower than that of the KP&F8 group (153.2 ± 69.50 N, *p* < 0.05; Fig. 5a), with the median of the KP&F8 group exceeding that of the KP group by 57.5%. Similarly, stiffness was significantly lower in the KP group (18.14 ± 9.84 N/mm) than in the KP&F8 group (38.25 ± 23.49 N/mm; *p* < 0.05; Fig. 5b), with the median of the KP&F8 group exceeding that of the KP group by 110.9%.

**Table 1:** Maximum fracture force (N) and stiffness (N/mm) of the specimens

	KP		KP&F8	
	Max fracture force	Stiffness	Max fracture force	Stiffness
Median	97.30	18.14	153.20	38.25
IQR	29.70	9.84	69.50	23.49
Mean	94.00	20.66	164.69	34.07
SD	17.41	5.78	47.53	12.39
Max	120.40	31.88	251.20	53.80
Min	63.30	14.98	94.30	15.70
Unit	N	N/mm	N	N/mm

*IQR* interquartile range, *SD* standard deviation, *KP* two K-wire group, *KP&F8* two K-wire with figure-of-eight cerclage wire group

In addition, the coefficient of variation (i.e., the standard deviation divided by the mean) of the maximum fracture force and stiffness in the KP and KP&F8 groups was 18.52 and 28.00% and 28.86 and 36.36%, respectively.

## Discussion

Metacarpal shaft fractures are a common type of hand fracture. Despite providing strong fixation strength, plate fixation has drawbacks. Concerning internal fixation with K-wires, although it is frequently used to treat metacarpal shaft fractures [8, 9, 24], the lack of reliable fixation strength in these wires may cause fixation failure. In the present study, figure-of-eight cerclage wire was used to assist K-wire fixation for metacarpal shaft fractures. This combined approach led to significant improvements in maximum fracture force and stiffness relative to K-wire fixation alone. To enhance fixation strength, when K-wire fixation alone cannot provide sufficient fixation strength, hand surgeons are advised to include a figure-of-eight cerclage wire in K-wire fixation for metacarpal shaft fractures.

Metacarpal shaft fractures are third only in frequency to distal radius fractures and phalangeal fractures [25]. reported that metacarpal shaft fractures account for approximately 18% of hand fractures. According to [26], 70% of all metacarpal shaft fractures occur between the second and fifth decades of life and are often caused by trauma or sports injury. Given this background, the loss of hand function among this population can result in substantial medical costs and loss of working hours [25, 27, 28]. Nonoperative intervention is applicable in the majority of cases of isolated metacarpal fractures [4, 29]. For every 2 mm of fracture shortening, there is approximately 7 degrees of extensor lag [30, 31]. Given that the metacarpophalangeal joint can hyperextend by approximately 20 degrees, shortening of up to 5 to 6 mm can be tolerated with the metacarpophalangeal joint in neutral [4]. The tolerable angulation in the sagittal plane—that is, both apex volar and apex dorsal angulation—varies for each metacarpal. For example, the index and middle finger metacarpals can tolerate up to 10 degrees of angulation, whereas the ring and little finger metacarpals can tolerate up to 25 and 45–50 degrees, respectively [32]. Scissoring deformity of the grasping fingers, which is caused by malrotation, has the limited range of tolerance. Specifically, when a finger crosses over or hits a neighboring finger as a result of a malrotation deformity of > 10 degrees, surgical treatment must be arranged [26]. Advances in surgical technique have contributed to the increasing use of surgical interventions for metacarpal fractures over the past two decades [24, 33, 34]. The present discussion of differences in outcomes between the two approaches (i.e., fixation using K-wires alone and fixation with both K-wires and figure-of-eight cerclage wire) can serve as a reference for the development of surgical strategies and the planning of postsurgical rehabilitation programs.

Owing to the challenges in obtaining real human metacarpals and the even greater difficulties in acquiring a sufficient number of specimens with similar bone strengths, artificial metacarpal shafts were used in the experiments, as suggested by Elfar et al. (2014) and in accordance with the [35] standard specification published by ASTM International. The experiments conducted by Barr et al. (2013), Watt et al. (2015), and Chiu et al. (2018) were used for reference, and cantilever bending tests were selected as the present evaluation method. On the basis of existing evidence [3, 15, 22, 36, 37], maximum fracture force and stiffness were selected as indicators of fixation strength. In our previous study on metacarpal neck fractures [15], stiffness referred to the stiffness of the fixation structure and the healing rate following fixation. Considering that recurrent metacarpal shaft fractures from high active or passive force are unlikely to occur, the assessment of stiffness is more relevant than that of maximum fracture force.

Among methods for fracture fixation that have been more frequently used in recent years, locking plate fixation provides greater fixation strength, which leads to faster healing of extremity fractures, enabling patients to regain their mobility and begin rehabilitation earlier. Locking plate fixation shortens healing time and accelerates the recovery of joint range of motion [38]. However, because the dorsal metacarpal skin is thin and the extensor digitorum tendon is closely attached to the bone, locking plate fixation has a higher incidence of complications such as metacarpophalangeal joint stiffness and extensor tendon adhesion. Such discomfort in the fracture site warrants secondary surgery for plate removal after the bone heals [4, 13]. Intramedullary screw fixation, an emerging surgical approach for fixating metacarpal shaft fractures, involves the retrograde implantation of a headless screw through the articular surface of the metacarpal head after fracture reduction. Because it does not involve contact with the extensor tendon, this method prevents extensor tendon adhesions, a sequela of plate fixation. However, the long-term impacts of cartilage damage caused by intramedullary screw fixation on joint movement require further investigation.

Recent studies have demonstrated that obsolete devices can ensure stable fixation in treating upper extremities fractures, with minimal invasiveness and low cost [39–41]. K-wire fixation, the most affordable type of surgical fixation, requires less soft tissue dissection than plate fixation does [42], thereby reducing the occurrence of extensor tendon adhesion. The drawbacks of K-wires include poor resistance to volar angulation deformity as well as fracture rotation, metacarpal shortening, wire migration, pin tract infection, and prolonged immobilization [43, 44]. Nevertheless, K-wires remain widely used in metacarpal fracture fixation because of their lower cost and minimal invasiveness.

The stiffness values in the KP and KP&F8 groups were 18.14 ± 9.84 and 38.25 ± 23.49 N/mm, respectively, greater than the corresponding values observed in our previous experiment on metacarpal neck fractures ([15] 16.9 ± 3.0 and 20.1 ± 3.2 N/mm, respectively). This might be ascribed to between-study differences in the type of fracture examined. An alternative explanation is the use of artificial metacarpal shafts, which we did not use in our previous study, in the present study. Despite the fact that Jones et al. (2019) used the same materials as the present study—that is, artificial metacarpal shafts and two K-wires—for metacarpal neck fracture fixation, their values of maximum fracture force (mean ± standard deviation [SD] = 279.7 ± 100.3 N vs. 153.20 ± 69.50 N) and stiffness (mean ± SD = 5.8 ± 0.5 N/mm vs. 38.25 ± 23.49 N/mm) differed from those we observed. This discrepancy can be mainly attributed to between-study variations in loading modality. As mentioned, metacarpal neck fractures were the focus of both our previous study ([15] and that by [16]. In clinical settings, fixating metacarpal shaft fractures with two K-wires is more challenging than fixating metacarpal neck fractures because the fractured end is farther away from the entry point of the wires and the trajectory of K-wires is more oblique. This means that accurate K-wire placement through minimally invasive percutaneous surgery is extremely difficult. Furthermore, incorrect placement can greatly reduce the fixation strength.

In clinical settings, the application of sufficient fixation strength to the fracture site enables earlier initiation of rehabilitation, thereby reducing both daily life inconveniences and economic losses (e.g., absence from work). As mentioned, maximal fracture force and stiffness were significantly greater in the KP&F8 group than in the KP group (*p* < 0.05). The use of figure-of-eight cerclage wire increased the maximum fracture force and stiffness by 57.5 and 110.9%, respectively, of that achieved in standard two K-wire fixation. Therefore, hand surgeons are advised to use the proposed approach to increase fixation strength in such procedures. Notably, the coefficient of variation was higher in the KP&F8 group than in the KP group, suggesting that the combined approach requires more surgical experience.

The in vitro biomechanical experiment confirmed that the figure-of-eight cerclage wire increased the ability of using two K-wires to fix transverse metacarpal shaft fractures. This technique provides an effective option for younger or high-demanding patients who may require a stronger fixation device. However, future clinical investigations should consider the following aspects. First, when using figure-of-eight cerclage wires to treat metacarpal bone fractures, the wire knot should be tied on the lateral side of the metacarpal bone. Subsequently, the wire knot should be moved to the lateral volar aspect of the metacarpal bone in order to keep the wire away from the extensor tendon and thereby prevent tendon injury. In brief, the extensor tendon must be treated gently during the procedure to prevent iatrogenic injury. The proposed technique is not more challenging than metallic plate fixation for metacarpal fractures and can be mastered through practice. Second, no studies have indicated the appropriate degree of fixation stiffness that can enable optimal treatment of metacarpal bone fractures. Studies have reported favorable outcomes in the use of K-wires alone for the fixation of metacarpal fractures. However, the stability of K-wire fixation remains questionable. On the basis of biomechanics and the present results, we recommend that the surgeon add a figure-of-eight cerclage wire if they have concerns regarding the stability of K-wire fixation in treating unstable metacarpal bone fractures.

This study has several limitations. First, owing to the difficulties in obtaining real bone, we had to use artificial metacarpal shafts instead. Second, the loading modality, specifically the cantilever bending tests used to compare the fixation strength attained under the two treatments, was unable to accurately simulate the forces applied to actual finger phalanges and metacarpals. Third, the combined method assessed in the present study was not examined or compared with other methods, such as plate fixation and headless screw fixation. Finally, because this was an in vitro biomechanical study, the effect of figure-of-eight cerclage wires on soft tissue, such as tendon or muscle, was not addressed. To gain a deeper understanding of metacarpal shaft fracture fixation, the clinical application of a figure-of-eight cerclage wire with two Kirschner wires, as done in the proposed procedure, should be further explored.

## Conclusion

In the present experiments, in which transverse metacarpal shaft fractures were made in artificial bone, the incorporation of a figure-of-eight cerclage wire into two K-wire fixation resulted in maximum fracture force and stiffness that were respectively 57.5 and 110.9% greater than those in the bones fixated with two K-wires alone. An in vivo study will be conducted to further explore the effect of the use of a figure-of-eight cerclage wire with two Kirschner wires, as done in the proposed procedure, before recommending it for clinical practice.

## Data Availability

All data generated or analyzed during this study are included in this published article.
